# Retrograde aortic access for ventricular arrhythmia ablation: A novel plug and suture strategy for vascular closure

**DOI:** 10.1016/j.hroo.2025.02.009

**Published:** 2025-02-21

**Authors:** Rebecca Arnet, Sven Knecht, Behnam Subin, Philipp Krisai, Nicolas Schärli, Felix Mahfoud, Christian Sticherling, Michael Kühne, Patrick Badertscher

**Affiliations:** 1 Department of Cardiology, University Hospital Basel, Basel, Switzerland; 2 Cardiovascular Research Institute Basel, University Hospital Basel, Basel, Switzerland

**Keywords:** Ventricular arrhythmia, Retrograde access, Vascular closure device, VCD, Suture based, Plug based

**Key Findings**

▪ Vascular access complications are frequently observed after catheter ablation of ventricular arrhythmias. Achieving hemostasis may be challenging after large-bore vascular access for retrograde aortic access.
▪ A novel technique combining a plug-based with a suture-based vascular closure device has been described outside of the catheter ablation population.
▪ In this study, the combination of plug- and suture-based vascular closure devices was feasible and appeared safe to achieve hemostasis in patients undergoing catheter ablation of ventricular arrhythmias using retrograde aortic access.
▪ Further studies assessing a larger patient population and cost-effectiveness are warranted.

The retrograde aortic (RA) approach is frequently utilized for catheter ablation targeting ventricular arrhythmia (VA). Various sheath types may be used for RA access, including deflectable and nondeflectable sheaths with larger diameters. Deflectable sheaths offer an additional pivot point, potentially enhancing stability and contact in regions with challenging catheter positioning, especially with irregular anatomy.

However, registry studies have demonstrated that the risk of complications during VA ablations is substantial, with a significant proportion attributable to vascular access.^1^ Consequently, there is a need for novel hemostasis methods following sheaths removal, especially when using deflectable sheaths with large-bore access, to reduce vascular complications and improve patient outcomes.

Current options for achieving hemostasis following femoral arterial access include manual compression and the use of vascular closure devices (VCDs). Insights from structural heart interventions, such as transcatheter aortic valve replacement, have contributed to advancements in safe RA access techniques. Recently, a novel technique combining a plug-based with a suture-based VCD has been described.^2^ This novel VCD strategy has not been tested in the VA ablation population, in which anticoagulation is uninterrupted. This study aimed to evaluate the safety and efficacy of the novel VCD strategy for large-bore vascular access following transfemoral VA ablation. The study was approved by the local ethics committees and complied with the Declaration of Helsinki. All patients provided written informed consent.

**Figure 1:**
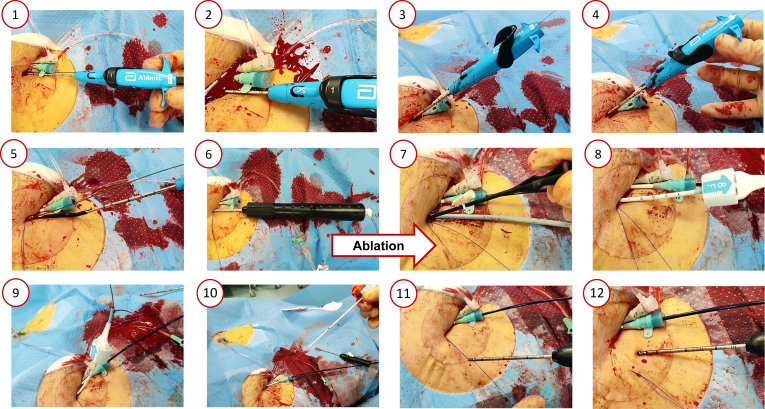
(1) Apply guide wires and introducers. Advance the suture-based vascular closure devices over the wire until the guide wire exit port aligns with the skin level. (2) Remove the guidewire, then advance the device further until artery blood is observed in the marker lumen. (3) Lift the lever to open the foot, stabilize the device at 45°, and depress the plunger by putting the right thumb on the handle and pulling with two fingers to deploy the needles. Cut the suture. (4) Lower the lever to close the foot. (5) Withdraw the device until the guidewire port is visible above the skin. Reintroduce the guide wire to maintain access and pull both suture limbs from the proximal guide. Remove device while keeping the guide wire in place. (6) Insert the steerable sheath and perform the ablation. (7) Load a snared knot pusher and advance the knot by applying slow consistent tension coaxial to the tissue tract. (8) Insert the guidewire and remove the procedure sheath. Advance until arterial blood flows from the drip hole in the locator. (9) Remove the arteriotomy locator and guidewire from the insertion sheath. Ensure that the tab on the reference indicator is in rear holding position and insert the reference indicator. Pull back the device cap until resistance is felt and the colored bands are visible. Lock the cap in place and remove the entire device. (10) Grip the appearing tamper tube to advance the knot and collagen while maintaining tension on the suture. When resistance is felt and hemostasis is achieved, cut the suture blow the stop on the suture. Remove the tamper tube and cut the suture again below skin level by pressing down the skin. (11) Pull back the thumb knot to open the suture gate at the distal end of the suture trimmer. Load one suture into the gate and advance the knot with the trimmer. Pull on the white-tipped nonrail to secure the suture knot tightly. Retract the trimmer. Pull back the thumb knot again and load both suture limbs into the gate and advance the trimmer. (12) Pull the red lever to trim the suture limbs.

Patients undergoing catheter ablation for VA using an RA at the University Hospital Basel were enrolled retrospectively. Arterial access was obtained via the right common femoral artery using ultrasound guidance. A novel plug- and suture-based VCD strategy following transfemoral VA ablation was applied as illustrated in Figure 1. Briefly, a suture-based VCD (ProStyle; Abbott Vascular) was inserted prior to the procedure and deployed at the 2 o’clock position. Following completion of the procedure, the introducer sheath was removed, and the suture-based technique was employed with a wire left in situ. Finally, a plug based VCD (Angio-Seal, 8F; Terumo Medical Corporation) was applied. No manual compression was used.

Vascular and access site–related complications, categorized as major or minor according to updated Valve Academic Research Consortium-3 criteria,^3^ were assessed through 30-day follow-up. The study was approved by the local ethics committees and adhered to the Helsinki Declaration.

A total of 17 patients were included (median age 63 [interquartile range 56–67] years; 35% female, median left ventricular ejection fraction was 47% [interquartile range 40%–56%]). All cases were performed using a steerable sheath (Agilis NxT, large curl; Abbott) via femoral RA, and in all cases the combined VCD strategy for vascular access closure was applied. Among the 17 patients, 9 (53%) patients underwent ventricular tachycardia, 7 (41%) underwent premature ventricular complex ablation, and 1 (6%) underwent combined premature ventricular complex/ventricular tachycardia ablation. The most common ablation target was the infero-latero-basal left ventricle. The median procedure time and ablation time were 205 (interquartile range 187–241) minutes and 1202 (interquartile range 661–1629) seconds, respectively. No major vascular complications occurred. Bedrest (6 hours) was extended for 2 (12%) patients due to puncture site bleeding, with 1 involving the venous puncture site. These cases could be managed conservatively using a pressure dressing. Small hematomas, not requiring intervention, were noted in 4 (24%) patients. No cases resulted in prolonged hospitalization, blood transfusions, or death. Protamine was used in none of the patients.

Recently, Tabaja and colleagues showed lower rates of vascular-related complications with VCDs compared with manual compression in patients undergoing VA ablation.^4^ Similar findings were found outside the VA ablation population with reduced time to hemostasis and earlier ambulation with the use of VCDs.^5^ The combination of plug- and suture-based VCDs may provide a more effective method for achieving hemostasis in large-bore access, addressing limitations of each individual device. Plug-based VCDs are limited to a sheath size of 8F. For large-bore arteriotomies, usually 2 suture-based VCD are required, increasing the risk of deployment failure and restrictions in terms of reaccess within specific time frames. Because many VA ablation patients are at increased risk of access site bleeding due to uninterrupted anticoagulation, the use of a VCD to achieve hemostasis is especially compelling in this population. Further studies assessing a larger patient population and cost-effectiveness are warranted.

Limitations include the single-center design, small sample size, and absence of a control group.

In conclusion, in patients undergoing VA ablation via RA access vascular closure using a combination of suture- and plug-based VCD is feasible and appears safe in the short term.
